# Virucidal efficacy of peracetic acid for instrument disinfection

**DOI:** 10.1186/s13756-017-0271-3

**Published:** 2017-11-10

**Authors:** Britta Becker, Florian H. H. Brill, Daniel Todt, Eike Steinmann, Johannes Lenz, Dajana Paulmann, Birte Bischoff, Jochen Steinmann

**Affiliations:** 1 Dr. Brill + Partner GmbH Institute for Hygiene and Microbiology, Norderoog 2, DE-28259 Bremen, Germany; 2 Institute for Experimental Virology, TWINCORE Centre for Experimental and Clinical Infection Research; a joint venture between the Medical School Hannover (MHH) and the Helmholtz Centre for Infection Research (HZI), Hannover, Germany; 3 Chemische Fabrik Dr. Weigert GmbH & Co.KG, Hamburg, Germany

**Keywords:** Peracetic acid, Virucidal efficacy, Instrument disinfection

## Abstract

**Background:**

Various peracetic-acid (PAA)-based products for processing flexible endoscopes on the market are often based on a two-component system including a cleaning step before the addition of PAA as disinfectant. The peracetic acid concentrations in these formulations from different manufacturers are ranging from 400 to 1500 ppm (part per million). These products are used at temperatures between 20 °C and 37 °C. Since information on the virus-inactivating properties of peracetic acid at different concentrations and temperature is missing, it was the aim of the study to evaluate peracetic acid solutions against test viruses using the quantitative suspension test, EN 14476. In addition, further studies were performed with the recently established European pre norm (prEN 17111:2017) describing a carrier assay for simulating practical conditions using frosted glass.

**Methods:**

In the first step of examination, different PAA solutions between 400 and 1500 ppm were tested at 20 °C, 25 °C, and 35 °C with three test viruses (adenovirus, murine norovirus and poliovirus) necessary for creating a virucidal action according to the European Norm, EN 14476. A second step for simulating practical conditions based on prEN 17111:2017 followed by spreading a test virus together with soil load onto a glass carrier which was immerged into a peracetic acid solution. A fixed exposure time of five minutes was used in all experiments.

**Results:**

In the quantitative suspension test 1500 ppm PAA solution was needed at 35 °C for five minutes for the inactivation of poliovirus, whereas only 400 ppm at 20 °C for adeno- and murine norovirus were necessary. In the carrier assay 400 ppm peracetic acid at 20 °C were sufficient for adenovirus inactivation, whereas 600 ppm PAA were needed at 25 °C and 35 °C and 1000 ppm at 20 °C for murine norovirus. A PAA solution with 1000 ppm at 35 °C was required for complete inactivation of poliovirus. However, a dramatically decrease of titer after the drying and immerging could be observed. In consequence, a four log reduction of poliovirus titer could not be achieved in the carrier test.

**Conclusion:**

In summary, 1500 ppm PAA at 35 °C was necessary for a virucidal action in the quantitative suspension test. After passing the requirements of the suspension test, additional examinations with adeno- and murine norovirus on glass carriers based on prEN 17111:2017 will not additionally contribute to the final claim of an instrument disinfectant for virucidal efficacy. This is due to the great stability of poliovirus in the preceded quantitative suspension test and the fact that poliovirus could not serve as test virus in the following carrier assay.

## Background

Peracetic acid (PAA) is often incorporated as active ingredient of instrument disinfectants for reprocessing flexible endoscopes in manual and automatic procedures. Such instrument disinfectants are often used between room temperature and 40 °C with short exposure times. By introducing PAA as active ingredient, a broad range of virucidal efficacy for instrument disinfectants can be achieved, as requested by the Commission for Hospital Hygiene and Infection Prevention (Kommission für Krankenhaushygiene und Infektionsprävention, KRINKO) [1]. There is only a minor temperature stress for the instruments when using short exposure times with PAA and only aldehydes are able to demonstrate a comparative range of efficacy against viruses. But for aldehydes, higher temperatures are necessary in general for reaching a sufficient virucidal action resulting in a claim of these chemicals against enveloped and non-enveloped viruses.

The virus-inactivating properties of PAA had been demonstrated earlier in detail by the group of Sprößig [2, 3]. Later it was questioned whether peracetic-acid-based formulations are suited for the cleaning step when reprocessing flexible endoscopes due to the fixation potential of PAA [4]. Current formulations on the market are always based on a two-step procedure including a cleaning step before the addition of PAA.

The concentrations of PAA in the products for reprocessing endoscopes differ and there are only few data on the behaviour of PAA in test methods developed as European Norms (EN). Therefore, we evaluated the virucidal activity of PAA solutions in clean conditions according to a quantitative suspension test (phase 2/step 1) which is described as EN 14476 with a short exposure time [5]. This was followed by a phase 2/step 2 carrier test, simulating practical conditions recently established as prEN 17111:2017 for instrument disinfectants in Europe [6].

## Methods

For the examination of the virucidal efficacy of different concentrations of PAA a quantitative suspension test according to the European Guideline EN 14476 with poliovirus (PV), adenovirus (AdV) and murine norovirus (MNV) as surrogate of human norovirus was used [5]. Subsequently, a quantitative carrier assay using frosted glass based on prEN 17111:2017 [6] was run with identical conditions regarding exposure time and test temperature with AdV and MNV and PV [6]. For all tests, clean conditions (0.3 g/L bovine serum albumin) and a fixed exposure time of five minutes were used.

PAA was supplied by AppliChem GmbH (order number 143495, 15% solution) (Ottoweg 4, DE-64291 Darmstadt). Dilutions of PAA were prepared with hard water according to the European norms immediately before the inactivation tests started.

PV type 1 strain LSc-2ab (Chiron-Behring) was obtained from PD Dr. O. Thraenhart, Eurovir, DE-14943 Luckenwalde. AdV type 5 strain Adenoid 75 (ATCC VR-5) from PD Dr. A. Heim, Institute of Medical Virology, Hannover Medical School, DE-30625 Hannover. MNV S99 was obtained from PD Dr. E. Schreier at the Robert Koch-Institute (RKI) in DE-13302 Berlin (now available at the Friedrich-Loeffler-Institute Bundesforschungsinstitut für Tiergesundheit, Ile of Riems).

The test virus suspensions were prepared by infecting monolayers of the respective cell lines. The virus titers of these suspensions ranged from 10^8^ to 10^9^ TCID_50_/mL (tissue culture infectious dose 50). PV type 1 was propagated in BGM cells (buffalo green monkey kidney cell line; supplied by Prof. Dr. Lindl, Institute for Applied Cell Culture, DE-81669 München) in Dulbecco’s Modified Eagle’s Medium (DMEM) with 1 g/L glucose. AdV type 5 replication was performed in A549 cells (human lung epithelial carcinoma cells). The A549 cells originated from the Institute of Medical Virology, Hannover Medical School, DE-30625 Hannover and were cultivated in Eagle’s Minimum Essential Medium with Earle’s BSS (EMEM). MNV strain S99 was propagated in RAW 264.7 cells (a macrophage-like, Abelson leukemia virus transformed cell line derived from BALB/c mice, ATCC TIB-71) in DMEM with 1 g/L glucose.

Tests according to EN 14476 were run with PV, AdV and MNV as test viruses of the EN 14476 in clean conditions with a fixed exposure time of five minutes [5]. 20 °C, 25 °C and 35 °C were used as test temperatures. Hard water was added as a control instead of PAA and cytotoxicity was additionally determined by addition of hard water instead of virus suspension. Infectivity was stopped by immediate serial dilution with ice-cold medium according to the standards of the European Committee for Normalisation [5, 6]. Of each dilution, 100 μL were placed in eight wells of a sterile polystyrene flat bottomed 96-well microtiter plate containing 100 μL cell suspension. Cultures were observed for cytopathic effects (CPE) after 4–10 days of inoculation depending on the cell culture system used.

The virus titers were determined using the Spearman and Kaerber method [7, 8] and expressed as log_10_TCID_50_/mL with 95% confidence interval (CI). Titer reduction caused by the biocide is presented as the difference between the virus titer after defined contact time with the water control and the disinfectant and defined as reduction factor (RF). A reduction of infectivity of ≥4 log_10_ steps (inactivation ≥99.99%, RF = 4) is regarded as evidence of virucidal activity.

The quantitative carrier test based on prEN 17111:2017 was performed in clean conditions with AdV and MNV and additionally with PV [6]. The surface sandblasted frosted glass carriers (15 mm × 60 mm × 1 mm, manufacturer: Zell Quarzglas und Technische Keramik Technologie GmbH, DE-21502 Geesthacht) were prepared as described in the prEN 17111:2017. One volume of interfering substance was mixed with nine volumes of test virus suspension (virus inoculum); 50 μL of this virus inoculum were pipetted on the inoculation square of the carrier followed by drying [6].

Ten mL of the different PAA solutions in a cylindrical screw tube were placed in a water bath at the chosen test temperature. After the drying process had been finished, the inoculated carrier was immersed in the prepared PAA solution (or hard water as control). Immediately at the end of the exposure time the carrier was transferred into a second screw tube with medium and glass beads and mixed for 60 s. After five minutes a second mixture was started for 60 s. Virus titer was determined by end point dilution titration in microtiter plates. Of each dilution 100 μL were placed in eight wells of a sterile polystyrene flat bottomed 96-well microtiter plate containing 100 μL cell suspension. Cultures were observed for cytopathic effects (CPE) after 4–10 days of inoculation depending on the cell culture system.

As in the suspension assay the method of Spearman and Kaerber [7, 8] was used for calculating virus titers. These were expressed as log_10_TCID_50_/mL with 95% CI. Titer reduction caused by the biocide is also presented as the difference between the virus titer after defined contact time with the water control and the disinfectant. As in the suspension test a reduction of infectivity of ≥4 log_10_ steps (inactivation ≥99.99%, RF = 4) is regarded as virucidal activity.

Linear regression analyses and statistical testing of differences between slopes and Y-intercepts were performed using GraphPad Prism v7.03. For individual linear regression per temperature, infectivity values between 400 and 1500 ppm PAA were taken into account (****P < 0.0001, **P < 0.01). Slopes and Y-intercepts are depicted in separate plots with 95% CI indicated by vertical lines.

## Results

**Fig. 1:**
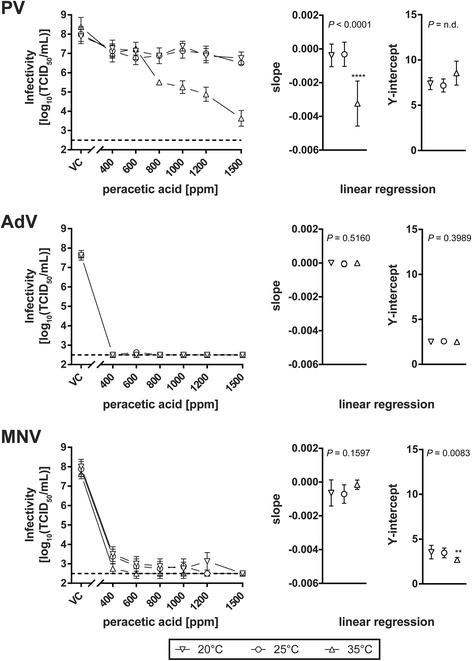
Inactivation of poliovirus (PV), adenovirus (AdV) and murine norovirus (MNV) by different peracetic acid concentrations at 20 °C (inverted triangles), 25 °C (inverted triangles) and 35 °C (triangles) in the quantitative suspension test depicted on the left as log_10_TCID_50_/mL. Exposure time was five minutes. The dotted line represents the detection limit of the assay. Slopes (± 95% CI) and Y-intercepts (± 95% CI) of respective linear regression analyses are shown in the right panels. ****P < 0.0001, **P < 0.01, n.d.n.d.*not determined* not determined

The PAA solutions between 400 ppm to 1500 ppm were examined in the suspension test according to the European Standard EN 14476 [5]. A four log_10_ reduction of the titer of PV was only achieved with 1500 ppm PAA at 35 °C when the initial titer of 8.38 log_10_TCID_50_/mL dropped to ≤3.63 log_10_TCID_50_/mL (RF = ≥ 4.75 ± 0.64) as depicted in Fig. 1. Lower concentrations (between 400 ppm and 1200 ppm) and lower temperatures (20 °C and 25 °C) were not successful in inactivating this respective test virus. These results were also visualized by a linear regression analysis and statistical testing of differences between slopes and Y-intercepts (Fig. 1). For inactivation of AdV, in nearly all cases no residual virus could be detected. The initial virus titre of 7.63 log_10_TCID_50_/mL at all temperatures tested decreased to ≤2.50 log_10_TCID_50_/mL (lower detection limit) resulting in a maximum RF of ≥5.13 ± 0.25. Likewise for MNV, 400 ppm PAA was able to inactivate the test virus at 20 °C. After five minutes exposure the titer was ≤3.50 log_10_TCID_50_/ml (initial virus titre 8.00 log_10_TCID_50_/mL, RF = ≥ 4.50 ± 0.52). In contrast to AdV, for all concentrations tested residual MNV could be detected except with 1500 ppm (Fig. 1).

**Fig. 2:**
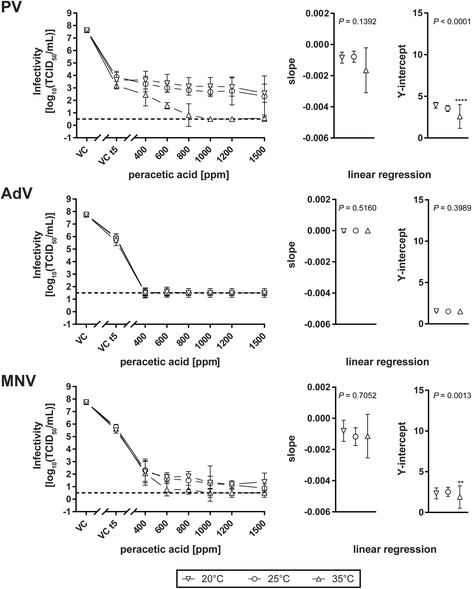
Inactivation of poliovirus (PV), adenovirus (AdV) and murine norovirus (MNV) by different peracetic acid concentrations at 20 °C (inverted triangles), 25 °C (inverted triangles) and 35 °C (triangles) in the carrier test depicted on the left as log_10_TCID_50_/mL. Exposure time was five minutes. The dotted line represents the detection limit of the assay. Slopes (± 95% CI) and Y-intercepts (± 95% CI) of respective linear regression analyses are shown in the right panels. ****P < 0.0001, **P < 0.01

In the test simulating practical conditions based on the prEN 17111:2017, the initial titer of PV dropped from 7.63 to 3.63 log_10_TCID_50_/mL at 20 °C, to 3.88 log_10_TCID_50_/mL at 25 °C and to 3.19 log_10_TCID_50_/mL at 35 °C in the virus controls, respectively, during the drying process and the additional incubation of the carrier in hard water (Fig. 2). Therefore, it was impossible with such a virus inoculum to demonstrate a four log_10_ reduction due to the virus loss. Nevertheless, no residual PV could be detected with 1000 ppm PAA at 35 °C. The initial virus titer of 3.19 ± 0.17 log_10_TCID_50_/mL dropped to ≤0.50 log_10_TCID_50_/mL (max. RF = ≥ 2.69 ± 0.17). For AdV 400 ppm PAA at 20 °C was sufficient for a four log reduction (Fig. 2). MNV was more stable than AdV in the carrier test requiring 1000 ppm PAA at 20 °C (RF = ≥ 4.19 ± 0.52) and 600 ppm PAA at 25 °C (RF = 4.13 ± 0.35) and 37 °C (RF = ≥ 4.87 ± 0.50) (Fig. 2).

## Discussion

There are some peracetic-acid-based products on the European market as recently listed by Kampf et al. [4] with different PAA concentrations and different exposure temperatures. They are used for instrument disinfection with a virucidal action presumably based on quantitative suspension tests. Currently, a European standardized test simulating practical conditions for reaching a virus inactivation is only being drafted with AdV and MNV as presumed test viruses [6]. PV is not included in this European normalisation assay due to problems of virus loss during drying.

In Europe, an instrument disinfectant has to pass first the quantitative suspension test followed by the carrier test. Therefore we used both test methods for evaluating the virus-inactivating properties of PAA.

Kline and Hull already demonstrated in 1960 for the first time, the strong virus-inactivating properties of PAA [9]. They showed that a 400 ppm PAA solution was able to produce a 7.5 log_10_ step reduction of PV after five minutes exposure time without any soil loading in a suspension test. Interestingly, they pointed out that formaldehyde showed an identical activity as a 5% solution after 20 min [9].

Additional data were published on solutions of PAA often in alcohol by Sprößig and Mücke [2, 3]. Furthermore, they introduced PAA as a disinfectant in human medicine [3]. The mechanism of PAA on viruses is characterised by disruption of the capsid and a RNA fragmentation as shown with PV type 1 [10].

Our data show that among the test viruses of the European norm EN 14476, the PV was much more stable than adenovirus and MNV that miss the strong hydrophilic character of PV. In contrast to the older data of Kline and Hull [9], higher concentrations were required but the experimental design between their study and our experiments is difficult to compare mainly related to the ratio of biocide to virus suspension and the soil loading.

Sauerbrei et al. found in comparative studies with PV type 1 and echovirus type 1 that 0.05% PAA was not active in clean and dirty conditions against PV, whereas 0.5% was virucidal within 10–30 min exposure time, thus showing similar data in comparison to our study [11]. For AdV type 5, Sauerbrei et al. found that 0.1% and 0.2% PAA were necessary to inactivate the AdV after 15 and 5 min, respectively [12]. But these tests were run with a higher soil load (10% foetal calf serum) according to the DVV Guideline in contrast to clean conditions.

In our suspension tests, AdV and MNV showed a similar behaviour. There might be a difference in stability between both viruses at concentrations lower that 400 ppm, However, we did not use lower concentrations because the concentration of PAA in the instrument disinfectant on the market in general is higher.

The greater stability of PV in contrast to AdV and MNV was also found performing test simulated practical conditions. Due to a high loss of virus titer for PV during drying and immerging, a four log_10_ reduction could not be observed with this virus. It can only be mentioned that 35 °C and at least 1000 ppm PAA were necessary to detect no residual PV. Here, in the test simulating practical conditions MNV was more stable than AdV. At 20 °C the required concentration of PAA for MNV was 1000 ppm in comparison to 400 ppm for AdV.

An identical procedure as shown here with frosted glass carriers was performed with PAA testing vaccinia virus strain Elstree and polyomavirus SV40 strain 777 by Strohhäcker und Eggers [13]. They found that even 0.05% PAA was sufficient for virus inactivation of both viruses within five minutes in clean and dirty conditions.

Following the procedure of virucidal testing in Europe, first the requirements of the suspension test have to be fulfilled. Then the phase 2/step 2 test must follow. According to our data with PAA and PV, it is much more difficult to reach an inactivation with PV, AdV and MNV in the quantitative suspension test due to the great stability of PV than to be successful with AdV and MNV in the carrier test based on prEN 17111:2017 simulating practical conditions. Therefore, it should be put into consideration in the future in Europe to include the stable murine parvovirus as a test virus in the phase 2/step 2 procedure even when using temperature < 40 °C. At the moment murine parvovirus is only used as sole test virus for instrument disinfectants at temperature ≥ 40 °C.

## Conclusion

In summary, 1500 ppm peracetic acid at 35 °C was necessary for a virucidal action in the quantitative suspension test. After passing the requirements of the suspension test, additional examinations with adeno- and murine norovirus on glass carriers based on prEN 17111:2017 will not additionally contribute to the final claim of an instrument disinfectant to have sufficient virucidal efficacy. This is due to the great stability of poliovirus in the preceded quantitative suspension test and the fact that poliovirus could not serve as test virus in the carrier assay.

## Abbreviation

AdV: Adenovirus
CI: Confidence interval
CPE: Cytopathic effect
DMEM: Dulbecco’s modified Eagle medium
EN: European Norm
KRINKO: Kommission für Krankenhaushygiene und Infektionsprävention
MNV: Murine norovirus
n.d.: Not determined
PAA: Peracetic acid
ppm: Part per million
prEN: European pre norm
PV: Poliovirus
RF: Reduction factor
RKI: Robert Koch-Institute
TCID50: Tissue culture infectious dose 50
